# Multi-hazard detection in the southern part of Banyuwangi Regency using a geomorphological approach

**DOI:** 10.4102/jamba.v16i1.1586

**Published:** 2024-07-30

**Authors:** Listyo Y. Irawan, Damar Panoto, Agus D. Febrianto, Vischawafiq Azizah, Siti N. Farihah, Muhammad Aufaristama, Mohammad T. Mapa

**Affiliations:** 1 Department of Geography, Faculty of Social Science, Universitas Negeri Malang, Malang, Indonesia; 2 Master Program of Educational Research and Evaluation, Graduate School, Universitas Negeri Yogyakarta, Yogyakarta, Indonesia; 3 Department of Applied Earth Sciences, Faculty of Geo-Information Science and Earth Observation, University of Twente, Enschede, Overijssel, The Netherlands; 4 Geography Programme, Faculty of Social Science and Humanities, Universiti Malaysia Sabah, Kinabalu City, Sabah, Malaysia

**Keywords:** multi-hazard, map, geomorphology, volcanic, coastal

## Abstract

Potential losses and damages caused by natural hazards in the future are essential information that the public and stakeholders need to understand. Banyuwangi Regency, located at the eastern most tip of Java Island, borders the Indian Sea on the south and the Bali Strait on the east. These areas are hazardous because they are at increased risks of severe weather with frequent occurrences of volcanic eruptions and tsunamis. The hazards and possibilities of the future can be detected through a geomorphological map. This map provides several details about the regional characteristics of Banyuwangi and information on the geomorphological configuration related to natural hazards and disaster-prone areas. Such information can be of use to the people, the government and stakeholders alike. Following the geomorphological approach in this study, we classified landforms using four aspects, such as (1) morphology; (2) morphogenesis; (3) morpho-arrangement and (4) morpho-chronology. The data sources used are geological maps, DEM Alos Palsar and Google Satellite imagery for interpretative analysis. In this study area, the landform mapping identified 15 different landforms. There are eight landforms with landslide hazards and three with tsunami hazards. The landform with a landslide hazard has a common morphological condition which is hilly morphology. The landform with tsunami hazards as the main triggering factor has a plain morphology and an elevation below 50 m above sea level. Those conditions allow the tsunami surge to run up to reach further areas until it reaches an undulating or hilly morphology.

**Contribution:**

The results of this research are expected to inform the multi-hazards sources based on the geomorphological conditions in the Banyuwangi Regency. With such information, the government and the people can increase their ability to cope with disaster strikes in the future.

## Introduction

Banyuwangi Regency has a very high tourism potential because of its diverse physiographical conditions, including volcanic, denudational, fluvial, marine, structural, karst and organic geomorphology. In general, the existence of these tourist destinations can improve the community’s welfare (Damanik 2013). The number of domestic and foreign tourists visiting Banyuwangi Regency has increased from 2016 to 2019. Total tourist visits in 2019 were 5 327 420. However, in 2020, it decreased to 2 594 977 on account of the coronavirus disease 2019 (COVID-19) pandemic. The increase in tourist visits from 2016 to 2019 cannot be separated from the various promotional activities and tourism events carried out by the Banyuwangi Regency Culture and Tourism Office (Betari Avinda Sudiarta & Oka Karini 2016; Kanom, Nurhalimah & Darmawan 2020).

For tourism to be sustainable, it is critical that governments invest in preparedness and disaster resilience (Chakraborty, Ibrahim & Cruz 2018). Therefore, there is an urgent need for a study related to the threat of disasters in Banyuwangi Regency considering that the region faces various disaster threats, such as volcanic eruptions, landslides, tornadoes, droughts, fires, tsunamis, floods and earthquakes (Faturahman 2020). Natural hazards can destroy people’s social lives, causing loss of life, damage to facilities and a decline in the quality and quantity of the victims’ livelihoods (Sawada & Takasaki 2017). A natural hazard can be broadly defined as a serious disruption to the functioning of a community or society that causes human, material, economic or environmental losses that extend and exceed the affected community’s ability to cope with disaster using its resources (UNISDR 2009).

This study identifies multiple disaster hazards through a geomorphological approach, focussing on the southern part of Banyuwangi Regency. Geomorphological conditions can represent a natural hazard in a certain landform (Chelli et al. 2021). This study aims to detect multiple disaster hazards in the southern part of Banyuwangi Regency, focussing on landslides and tsunamis. Geomorphology comprises a broad scope of studies, such as representation, analysis and visualisation of the shape of the Earth’s surface and the processes that occur in space (Bachri et al. 2021). The geomorphological approach is based on four aspects, namely morphology, morpho-arrangement, morphogenesis and morpho-chronology (Dahroni, Arozaq & Khoirunisa 2017; Santosa 2016; Van Zuidam & Van Zuidam-Cancelado 1979).

This research uses a Geographic Information System (GIS) geomorphologic approach for debris risk assessment. Baiocchi et al. (2023) adopting a geomorphological approach based on Digital Terrain Models (DTM) data analysed the risk and vulnerability of shallow landslides in Sicily, Italy using QGIS (previously known as Quantum GIS) applications, and slope and hillshade tools. However, this study also used terrestrial surveys to calculate soil thickness using the Green Infrastructure Space and Traits (GIST) model approach.

Another approach to disaster risk detection is the GIS-based morphotectonic analysis that delineates multi-hazard areas based on spatial analysis (Endyana et al. 2017). Disaster risk uses Digital Elevation Model (DEM) data and geological maps to analyse flood and landslide risks in the southeastern West Java province by taking into account slope and elevation values, dip-slip and strike-slip mechanisms, basins from DEM analysis and floodplains because of sea level rise of 5 m–30 m.

This research is inspired by the land resources landscape of the Ijen Volcano Complex and its surroundings (Sartohadi et al. 2014), which describes the general geomorphology of the Ijen Volcano. This area consists of several landforms, that is, structural, volcanic, solutional, fluvial, marine and denudational. Those classifications are based on landform genesis or the origin of the landform processes. Moreover, this research also reveals multi-hazard disasters generated from geomorphological conditions and processes that may trigger natural hazards in Ijen Volcano and its surroundings.

## Study area

**FIGURE 1:**
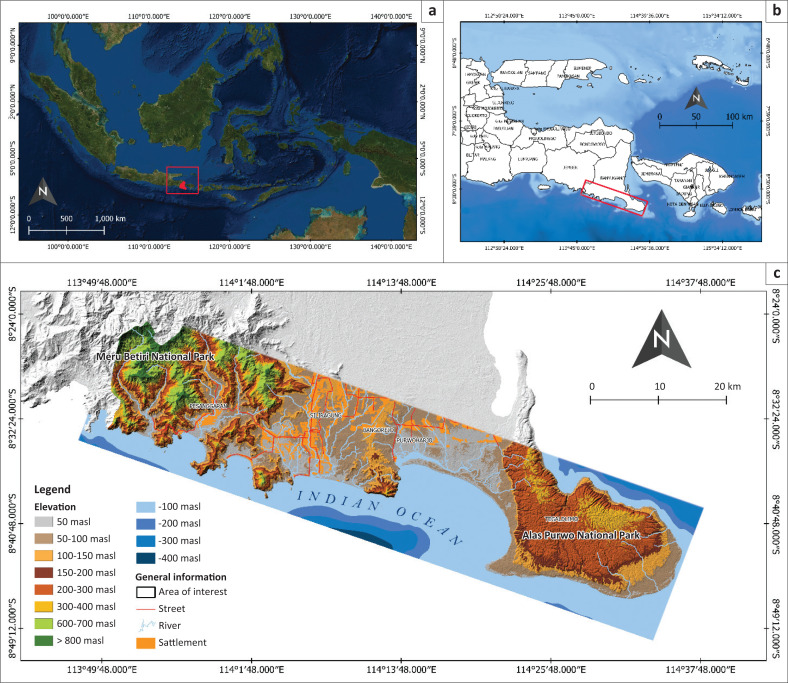
(a) Banyuwangi Regency in Indonesia, (b) research area in Banyuwangi Regency and (c) Close-up of the research area as part of the Banyuwangi Regency.

Banyuwangi Regency is located at the east end of Java Island (Figure 1). It faces the Indian Ocean in the South and Bali Strait in the east. Specifically, this study focussed on the southern part of Banyuwangi Regency, which includes five districts, namely: (1) Pesanggaran, (2) Siliragung, (3) Bangorejo, (4) Purwoharjo and (5) Tegaldimo. Siliragung has the highest settlement density followed by Bangorejo, Purwoharjo, Pesanggaran and Tegaldimo. This study area has two national parks: Meru Betiri National Park in the west and Alas Purwo National Park in the east (Blambangan Peninsula). It also has popular beaches as tourist destinations, such as Teluk Hijau, Rajegwesi, Wedi Ireng, Mustika Pancer, Pulau Merah, Lampon and Plengkung.

The study area covers an area of about 118 848.243 ha. Based on the topographical conditions, the study area has a maximum elevation of about 1200 masl (metres above mean sea level) in the complex of Meru Betiri National Park. The southern part of Banyuwangi area lies within a tropical monsoon climate zone with rainfall ranging from a minimum of around 20.88 mm in most dry months (September) to a maximum of 262.30 mm in rainy months (January). The average annual rainfall is 113.46 mm.

## Method

**FIGURE 2:**
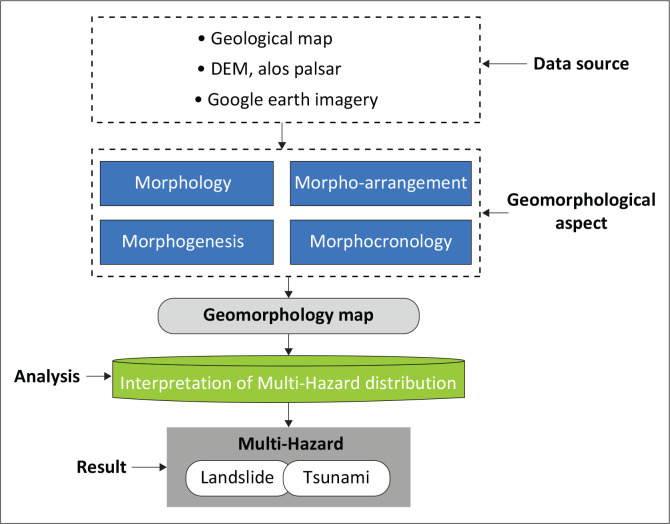
The stages of Geomorphological mapping.

This research mainly uses quantitative methods derived from maps and the GIS approach for analysis. Some works of literature and documents are used to describe the detailed condition of the research area. The authors also conducted detailed field surveys to evaluate the real surface condition. The flowchart in Figure 2 outlines the methodology used in this article.

As shown in Figure 2, the geomorphological mapping stage comprises several integral steps. First and foremost, the data utilised for this mapping process are derived from diverse sources, including geological maps, DEM from ALOS PALSAR and satellite imagery from Google Earth. These sources collectively contribute a comprehensive dataset, facilitating a thorough exploration of the landscape. The second stage involves the examination of various geomorphological aspects, namely morphology, morpho-arrangement, morphogenesis and morpho-chronology. This holistic approach enables a nuanced understanding of landforms, their spatial organisation, evolutionary processes and temporal sequences. The culmination of these efforts is manifested in the creation of a geomorphology map, a visual representation that encapsulates the intricate features and characteristics of the studied area.

Subsequently, the analysis phase delves into the interpretation of multi-hazard distribution. This involves a meticulous examination of the geomorphological map to identify and assess potential risks associated with multiple hazards. The emphasis is placed on understanding the distribution patterns of hazards, particularly focussing on the presence of threats such as landslides and tsunamis. The final result of this comprehensive mapping and analysis process is the identification and documentation of multi-hazard occurrences, highlighting the areas susceptible to various geological risks. This valuable information aids in informed decision-making for disaster mitigation and risk management strategies, providing a crucial foundation for safeguarding vulnerable regions against the potential impacts of landslides and tsunamis.

The source and criteria of the data are detailed in Table 1.

**TABLE 1:** Data type and source of data acquisition.

No	Data	Source	Version
1	Geological maps	Ministry of Energy and Mineral Resources through the website: https://geologi.esdm.go.id/geomap	Scale 1:100 000 sheets Banyuwangi; Jember and Blambangan
2	DEM ALOS PALSAR level 2.2	National Aeronautics and Space Administration (NASA) https://cmr.earthdata.nasa.gov/search/concepts/C2011599335-ASF.html	Temporal extend: 23 May 2011 Resolution 12.5 m
3	Google Earth imagery	XYZ tiles from QGIS with URL Satellite imagery: https://mt1.google.com/vt/lyrs=s&x={x}&y={y}&z={z}	CRS: EPSG:3857–WGS 84 Render type: single-band colour data Resolution: 0.3 m–1.2 m

*Source:* Ministry of Energy and Mineral Resources, accessed from https://geologi.esdm.go.id/geomap; NASA, accessed from https://cmr.earthdata.nasa.gov/search/concepts/C2011599335-ASF.html; and XYZ tiles from QGIS, accessed from https://mt1.google.com/vt/lyrs=s&x={x}&y={y}&z={z}

**FIGURE 3:**
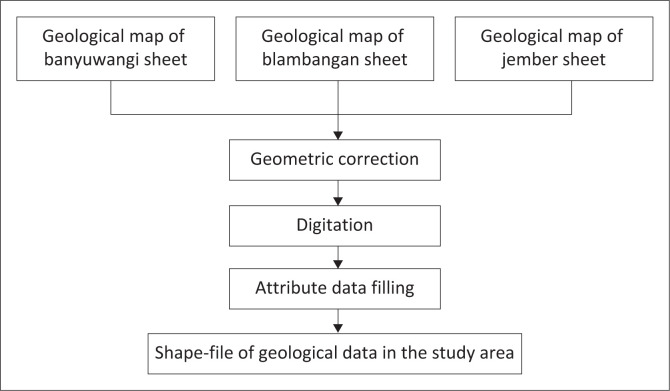
Diagram of a geological map’s process flow.

A geological map with a scale of 1:100 000 was chosen to provide easy interpretation of the data. The Indonesian Ministry of Energy and Mineral Resources provides geological data in various versions. They are 1:100 000, 1:250 000, 1:500 000, 1:5 000 000 and 1:1 000 000 scales in printed map form or JPG format so that one administrative area can be divided into several sheets. To obtain clear and complete data on the study area, three geological map sheets: the Banyuwangi, Blambangan and Jember sheets are needed. To collect and analyse this data, firstly, we geometrically correct it and then digitise it according to the shape of the map (see Figure 3).

The SHP (shapefile) results on the combined geological map are then visualised using categorised symbols in the QGIS and are interpreted manually. The basis for data collection is already contained in the geological map and listed on the results and discussion section map. The second data source is the Alos Palsar DEM data. The DEM is used to analyse the slope elevation and slope to be discussed in the morphology, morphogenesis and morpho-arrangement sections further in the article.

Data obtained from the NASA official website also has an adequate resolution to show the area’s relief with the required area of about 118 848.243 ha. Data sources selected are the official website pages and can be freely accessed by the researchers. The third data source is the satellite imagery obtained directly from the QGIS base map using the XYZ feature. Satellite imagery data has a high enough resolution that is suitable as interpretation material in the study area.

The geomorphological condition was investigated using an integrated aspect incorporating: (1) morphology, (2) morphogenesis, (3) morpho-arrangement and (4) morpho-chronology. Details of the geomorphological mapping stages are presented in Table 2. The analysis used a wide range of imagery and theme maps across various scales. Geomorphological and topographic analyses were combined with field surveys to verify the potential hazard from all process of geomorphology mapping in this research with the GIS Software (QGIS 3.18). Geographic Information Systems can exploit digital datasets for multi-level geomorphological mapping, from fine to coarse (de Jong et al. 2021). The geomorphological mapping research uses a small scale of <1:250.000 (Van Zuidam & Van Zuidam-Cancelado 1979). The selection of this scale can reach the area studied with a fairly clear visualisation.

**TABLE 2:** Geomorphological mapping stages of Landform identification.

Stages	Source	Processing technique
Morphology	DEM ALOS Palsar, Resolution 12.5 m	Interpretation of relief visually/manually using slope analysis and hillshade (Van Zuidam & Van Zuidam-Cancelado 1979)
Morphogenesis	DEM ALOS Palsar, Resolution 12.5 m Geological Map Scale 1:100 000	Interpretation of landforms based on the origin of the process is carried out visually/manually through hue, colour, shape and texture in terms of the association of geological material composition (Van Zuidam & Van Zuidam-Cancelado 1979)
Morpho-arrangement	DEM ALOS Palsar, Resolution 12.5 m Geological Map Scale 1:100 000	Visual/manual interpretation using slope analysis, hillshade, profiling and review with the association of geological material composition
Morpho-chronology	Geological Map Scale 1:100 000	Reviewing geological and soil material information

*Source:* National DEM with ALOS PALSAR data at a resolution of 11.25 m, Geospatial Information Agency 2018, accessed from https://tanahair.indonesia.go.id/demnas/#/ and Geological Map Scale 1:100,000, Geological Survey Center, Map Data by GeoMap [V 2.2.1], accessed from https://geologi.esdm.go.id/geomap

At the morphological stage, slope and hillshade analysis was conducted through DEM ALOS Palsar. The tools used at this stage are slope and hillshade (tools in the raster menu in the QGIS application) with the slope provisions (Table 3).

**TABLE 3:** Relief classification according to Van Zuidam.

Relief classifications	Slope classifications (%)	Height
**Flat/almost flat**	0–2	< 5
**Undulating**		
a. Gently undulating	3–7	5–50
b. Undulating	3–7	5–75
c. Sloping undulating	8–13	5–25
**Undulating rolling**		
a. Undulating – rolling	8–13	25–75
b. Rolling	8–13	75–200
a. Moderately steep rolling	14–20	25–50
**Rolling – hilly**		
a. Rolling – hilly	14–20	50–200
b. Hilly	14–20	200–500
b. Steep hilly	21–55	50–200
**Hilly – steeply dissected**		
a. Hilly – steeply dissected	21–55	200–500
b. Steeply dissected	21–55	500–1000
c. Very steeply dissected	56–140	200–500
**Steeply dissected – mountainous**		
a. Steeply dissected – mountainous	56–140	500–1000
b. Moderately steep mountainous	56–140	> 1000
**Mountainous**		
a. Mountainous	> 140	500–1000
b. Extremely steep mountainous	> 140	> 1000

*Source*: Listyani, T.L.R., 2019, ‘Criticise of Van Zuidam classification: A purpose of landform unit’, *ReTII* 332–337, viewed 10 December 2023, from https://journal.itny.ac.id/index.php/ReTII/article/view/1432

The slope guidelines are also used in morphogenesis and morpho-arrangement analysis. Visual interpretation is done by distinguishing the slope based on colours symbolised using graduated symbols to see the slope level in the study area. In morphogenesis and morpho-arrangement analysis, the interpretation of geological maps with a scale of 1:100 000 is used to obtain clearer and more accurate data. The geological map that is the basis for morphogenesis, morpho-arrangement and morpho-chronology analysis can be accessed on the webpages listed in Table 1. The main geological table will show information on landform complex units, types of rocks that make up a landform complex, cross section and age of rocks according to stratigraphic rules as the basic material for analysis. As for profiling, use the profile tools plugin in QGIS software.

## Ethical considerations

An application for full ethical approval was made to the Universitas Negeri Malang Ethics Committee, Indonesia and ethics consent was received on 8 March 2024. The ethics approval number is 8.3.3/UN32.14/PB/2024.

## Results and discussion

### Geomorphology

**FIGURE 4:**
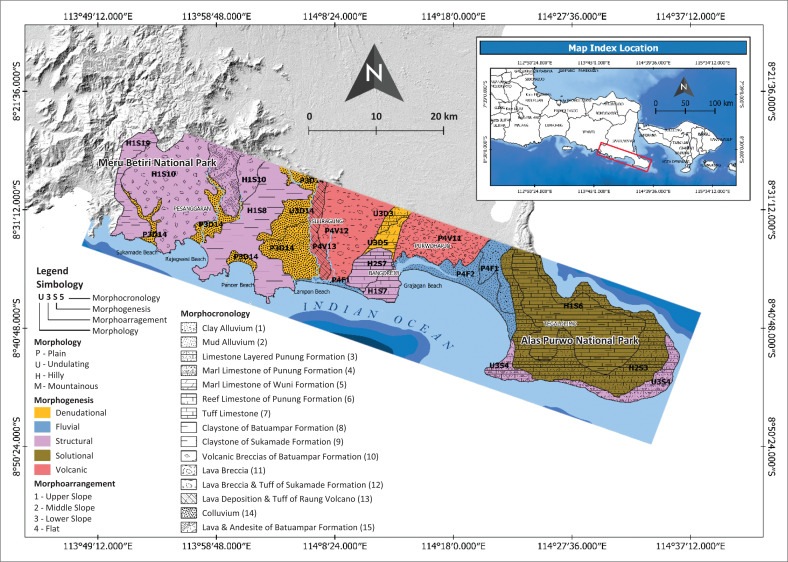
Geomorphological map of the southern part of Banyuwangi Regency.

This research through the geomorphological approach reveals and identifies the threatened areas based on the natural hazards. The geomorphological approach can be divided into four major aspects: (1) morphology, (2) morphogenesis, (3) morpho-arrangement and (4) morpho-chronology. Geomorphology not only gives information about the terrain condition but is more complex, defining its historical development and physical processes acting over given time in every landform unit (Gustavsson, Kolstrup & Seijmonsbergen 2006). Based on each of the four aspects that are used in this study, the historical development and physical process of each landform unit can be explained in a unique relationship. In this study area, the landform mapping identified 15 different landforms (Figure 4).

The morphological aspect is divided into four classes, namely: plain, wavy, hilly and mountainous (Van Zuidam & Van Zuidam-Cancelado 1979). Southern part of Banyuwangi Regency has the plain–hilly morphological condition. Plain morphologies are dominant in low elevation, about 50 masl. Plain morphologies are controlled by accumulation of material depositional from denudational, volcanic and fluvial processes. In more rough morphological conditions such as undulating and hilly dominant in structural process in complex of Meru Betiri and a few parts of Blambangan Peninsula steep coast, also in residual form from denudational process in southern of Meru Betiri complex. Blambangan Peninsula with solutional landform have a hilly morphology.

Figure 4 shows that the Pesanggaran subdistrict on the south side of the western part has several morphological forms including plains, wavy, to hilly. Some watersheds that flow in this area are the Karang Tambak and Gonggo watersheds. In addition, Meru Betiri National Park is a conservation area whose morphology is dominated by karst landscape, fluvial and organic marine vegetation in its coastal area which is included in the Pesanggaran district. In several locations, there are also many famous areas, including Sukamade Beach, Green Bay, Rajegwesi Beach, Muara Mbaduk Beach, Wedi Ireng Beach, Pancer Beach, Red Island and Lampon Beach.

Other areas on the southern side of the centre are Siliragung and Bangorejo sub-districts. The morphology of this area is dominated by plains, wavy and partly hilly. There are four watersheds that drain into this area, namely: Kali Baru, Putih, Dogong and Gedong. In addition, on the south side of the southeast part of Purwoharjo and Tegaldlimo sub-districts, there are at least five watersheds that drain into this area, including: Segoro Anakan, Bantenan, Karang Mente, Kapal Pecah and Slaka. In the easternmost part of the region, Alas Purwo National Park covers the entire Blambangan Peninsula and a small part of the coastal area in the south and east. Fluvial landforms, karst and organic marine vegetation in some coastal areas dominate the morphology of the Alas Purwo National Park conservation area.

In addition to the conservation area of Alas Purwo National Park, several locations in Purwoharjo and Tegaldlimo sub-districts in the south and southeast have been built with several facilities for various uses, such as tourism. Some locations developed for tourism are Grajagan Beach, Bedul Mangrove Area, Ngagelan Beach, Trianggulasi Beach, Savana Sadengan, Pancur Beach, Parang Ireng Beach and Plengkung Beach (G-Land). In the coastal area of Plengkung Beach (G-Land), which has one of the best waves in the world, many types of organic marine landscapes are identified, one of which is organic coral. In addition to organic coral formations, marine landscapes found in the coastal environment of the Blambangan Peninsula include beaches (sea sand) scattered along the coastline.

Banyuwangi Regency has five morphogenesis categories, namely: (1) denudational, (2) fluvial, (3) structural, (4) solutional and (5) volcanic. The origin process includes the main groups of endogenic (mainly constructional) and exogenic (mainly denudational) forces (Gustavsson et al. 2006). The denudational process can be identified easily through the high erosion and landslide phenomena (Hadmoko et al. 2010). The landform originating from the fluvial process is controlled by the activity of river water flow which generally has a sloping to flat topography with the general material being alluvium (Corenblit et al. 2007). Solutional landforms are controlled by the activity of dissolving limestone areas by water. Meanwhile, the landform originating from the volcanic process is controlled by volcanic activity.

The morpho-arrangement aspect is related to the spatial arrangement around the landform. It is divided into four classes, namely: (1) upper slope, (2) middle slope, (3) lower slope and (4) plain. The upper slope of the Banyuwangi Regency is dominated by the peak area of the Meru Betiri and the other part is located at the top of the karst hills of Alas Purwo National Park on the southeast side of Banyuwangi. The middle slope can be found between the transition from upper slope into lower slope. A few morphogenesis complexes in the Banyuwangi Regency that transition from the upper slope into the lower slope are not clearly visible. Thus, the middle slope is not visible like in the structural complex of Meru Betiri. The lower slopes can be found in the dominance of landforms from denudational processes and few on structural and solutional process, with regional characteristics that tend to be moderate to steep hills. The final morpho-arrangement condition is a plain where the landforms in the environment tend to be influenced by depositional of fluvial, denudational and volcanic process. This plain environment can be found in densely populated settlements, transportation/administrative centres and marine tourism in Banyuwangi Regency because its accessibility is relatively easier to reach.

The morpho-chronological aspect explains the sequence or history of landform formation. This information can be seen from the structure and geological material. The geological conditions of the Banyuwangi Regency are relatively the same as that of the island of Java. Banyuwangi Regency was formed from the tertiary era of the Early Miocene to the recent (quaternary) where the plate subduction process in the Indian Ocean is still moving with an intensity of 7 cm/year (Wang et al. 2019a). This plate subduction movement is one of the endogenous forces forming landform units which is also influenced by exogenous forces, better known as tectonic geomorphology. In essence, this tectonic geomorphology examines the relationship between the Earth’s internal (tectonic/endogenic) and external (surface/exogenic) processes.

### Multi-hazard

The main control for the high level of landslide hazards is morphological conditions which are primarily dominated by hilly-mountainous morphology. This condition is in line with the results of previous studies which prove that morphological conditions such as slope level are the most influential on landslide hazards (Ayalew et al. 2005; Irawan et al. 2021a; Pamela et al. 2018; Wang et al. 2019b). Morphology effects the slope instability either directly or indirectly, such as: (1) increasing or reducing the shear strength, (2) controlling the microclimatic parameters such as exposure to sunlight, wind, rainfall intensity and slope material properties, and (3) controlling the landscape forms (Conforti et al. 2014; Lombardo et al. 2015). The hilly-mountainous morphology has a high amount of flow and energy for the rate of water transportation, so it tends to be unstable and prone to landslides. This process is caused by an increase in gravity that is directly proportional to the steepness of the slope (Pradhan & Kim 2016).

Elevation also controls the frequency of landslide occurrences (Ayalew & Yamagishi 2005). Generally, landslide susceptibility is higher at intermediate and high elevations compared to low elevations because of the more gentle slopes at low elevations, whereas at intermediate and very high elevations, landslides have greater shear strength (Dai & Lee 2001, 2002). Past landslide hazards can be identified through the shape of the slope, such as the concave of the escarpment, landslide depositional material and open slope from land cover like vegetation in some steep slopes (Kornejady, Ownegh & Bahremand 2017; Nguyen et al. 2019).

**FIGURE 5:**
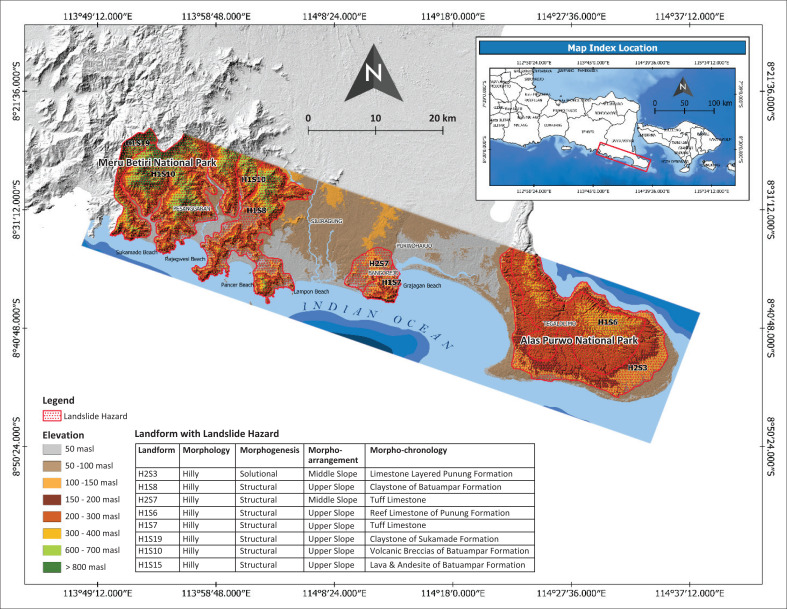
Map of landslide hazard in southern part of Banyuwangi Regency.

Based on landform classification, there are eight landforms with landslide hazards in southern part of Banyuwangi Regency (Figure 5). All such landforms have a common similarity in the morphological condition, which is hilly morphology. On the other hand, based on the morphogenesis aspect, the landform with landslide hazards was formed by structural process (Meru Betiri National Park and structural hills part in Bangorejo District) and solutional process (Alas Purwo National Park). Commonly, middle–upper slope morpho-arrangement has more steepness, thus increasing the gravity to triggering the slope failure (Chen et al. 2003).

Morpho-chronology indicated by geological material can control the landslide material and strength of the slope. Landforms with landslide hazards have various geological material, such as limestone, claystone, tuff limestone, reef limestone, claystone, volcanic breccias, lava and andesite. Landslide material is mainly composed of rock and soil (Varnes 1978), in comparison to the new definitions of landslide material based on genetic and morphological aspects rather than arbitrary grain-size limits. The basic material groups include sorted materials (such as gravel, sand, silt and clay) and unsorted materials (such as debris, earth and mud, peat and rock).

The 1994 tsunami in Banyuwangi Regency caused great damage; there were 200 deaths in total, spread across the Southeast of Java Island and Southwest of Bali (Synolakis et al. 1995). This tsunami was triggered by a 7.2-magnitude earthquake at a depth of 15 km near the east end of the Java trench in the Indian Ocean. Rajegwesi, Pancer, Pulau Merah, Lampon and Grajagan beaches were the hardest hit shores by this tsunami in the southeast Java (Irawan et al. 2021b; Synolakis et al. 1995), with maximum runup hits of the order of 12 m.

Underwater tectonic activity is the dominant factor that causes tsunamis (Satake et al. 2013). The tsunami’s damage impact is controlled by earthquake magnitude in the ocean and geomorphological features such as morphology (Murthy et al. 2006). Tsunami geomorphology looks at landforms and the processes that created them to identify the susceptible area. The most dangerous areas are coastal zones with gentle slope resulting in large inundation (Mahendra et al. 2011). Specifically, geomorphological conditions control tsunamis through five main variables: sand availability, embayment type, nature of the coast, accumulation space and landward environmental conditions (Goff, Lane & Arnold 2009).

**FIGURE 6:**
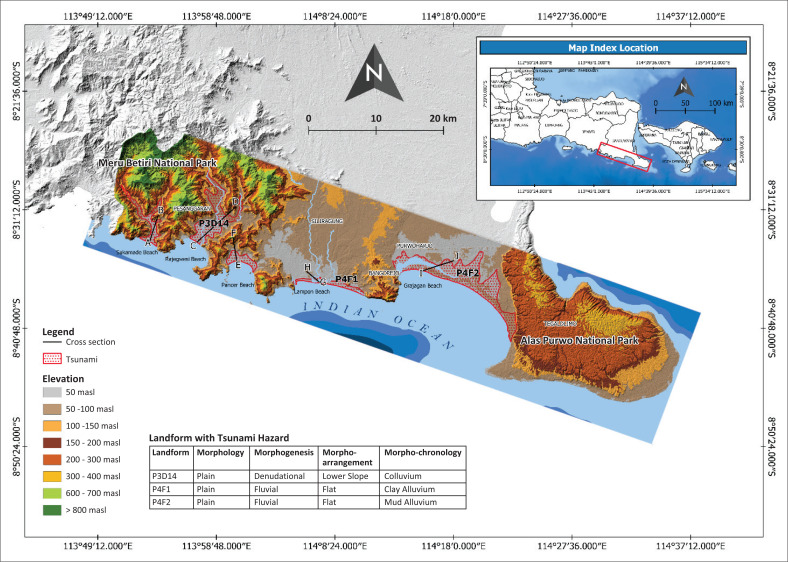
Map of tsunami hazard in the southern part of Banyuwangi Regency.

Landform units with a tsunami hazard include P314, P4F1 and P4F2 (Figure 6). Though the tsunami hazards affected the landform along the structural cliff in southern part of Banyuwangi Regency, it did not lead to greater economic losses and environmental damages or even victims because this structural cliff acted as a barrier. Based on landforms with tsunami hazards, this study has strong similarities, such as an elevation above 50 masl and no barrier. Figure 6 shows the landform area with tsunami hazard along with the view of several cross sections to help us identify the morphological features that played a big role in controlling the runup flow and the inundation area.

**FIGURE 7:**
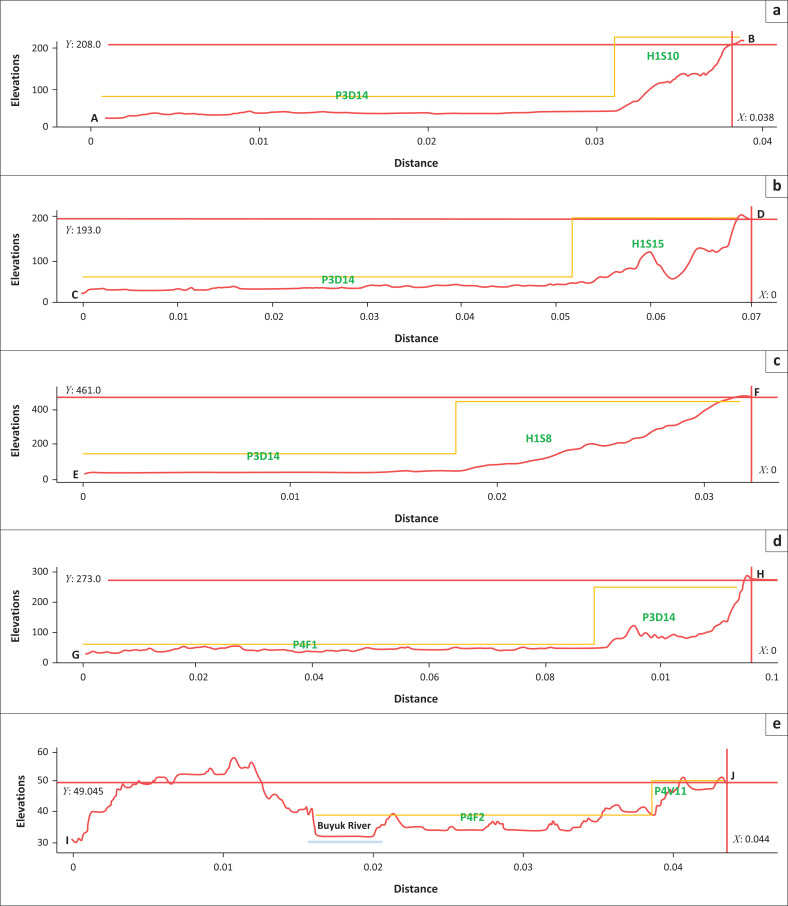
Cross section showing geomorphological feature: (a) Cross section A–B, (b) cross section C–D, (c) cross section E–F, (d) cross section G–H and (e) cross section I–J.

The cross section in Figure 7 shows the profile of geomorphological conditions in an area with tsunami hazard. Morphology in every landform with tsunami hazards can be easily seen. All characteristics in P3D14, P4F1 and P4F2 have plain morphology. Plain morphology allows the tsunami surge to run up to reach further areas until it cannot anymore because it is blocked by rougher morphology (undulating or hilly).

Cross sections a, b and c of Figure 7 show the morphological feature in P4D13 landform with tsunami hazard. This landform has an elevation of 50 masl and below, has a plain morphology, a denudational morphogenesis, is part of lower slope of Meru Betiri Mountain range and is structured by colluvium material. Colluvium are heterogenous materials of any particle size, generally composed of soil and/or rock fragments. This landform is a depositional place from denudational process in the middle slope–upper slope by gravitational process, soil creep, sheet flow, rain wash or mudflows.

Cross section d of Figure 7 shows the profile of P4F1 morphology and cross section e of Figure 7 shows the profile of P4F2 morphology. Those two landforms have similar characteristics, which is plain morphology, formed by fluvial process and on flat morpho-arrangement. On the other hand, both the landforms are structured by different materials – P4F1 is structured by clay alluvium and P4F2 by mud alluvium.

P4F2 landform has unique geomorphological features (Buyuk River and beach ridges) that are able to control the runup and flow of tsunamis. The river is able to play the role of a carrier and/or conveyor because tsunami surge can easily go inland and cause wide destruction up to considerable distance. Besides that, this landform also has beach ridges that can act as natural a barrier of tsunami surge. The capacity of barrier depends on the height of the barrier itself and the tsunami surge. These beach ridges have a maximum elevation of about 50 masl so they can turn out the tsunami surge around that height.

Each profile can be described in detail as follows: P3D14 is characterised by threats from the tsunami wave. The following location characteristics are derived from morphogenesis related to landslides because the landform genesis is from a denudational process. This process is mainly affected by weathering and erosional processes. In detail, the profile is characterised by different elevations and morphologies. P3D14 is characterised by plain morphology in denudational landforms. P4F1 and P4F2 are plain morphologies in fluvial areas. Hilly areas on structural landforms define H1S8, H1S10 and H1S15. Lastly, P4V11 is identified as undulating to hilly areas in the volcanic landforms.

Each landform has unique characteristics. In denudational landforms, the dominant process is weathering and erosion. It also works on solutional landforms. At the same time, structural landforms are dominated by tectonic activity. On fluvial landforms, erosion, transportation and sedimentation of material from volcanic areas are the most dominant. These findings are similar to a previous study conducted by Sartohadi et al. (2014), which found that the characteristics of landforms generated from GIS analysis on the slope, hillshade and geological conditions explain the current condition of the study areas. This analysis can help identify the potential natural hazards. The research analyses morphological conditions related to natural hazards, especially in coastal areas threatened by the tsunami wave.

## Conclusion

Natural hazards can be identified from the earth’s surface interpretation. This interpretation is known as the geomorphological approach. The key interpretation is the morphology of the research area. This research area has two main potential hazards from the geomorphological conditions: landslides and tsunamis. The landslide hazards can be identified from slope steepness found in hilly areas. The tsunami hazards mainly threaten the plain areas along the Indian Ocean, where the wave can reach locations below 50 masl. This research reveals that the geomorphological approach clarifies the potential hazards. The southern part of Banyuwangi Regency previously suffered from tsunami waves and another disaster strike. The research findings help us understand the detailed geomorphology of threatened areas affected by natural hazards. However, the geomorphological approach is unsuitable for more detailed research in natural hazard studies, such as building a probability of hazard class. The geomorphological approach depends on the researcher’s interpretation, so in some cases, different researchers may have different interpretations controlled by the cause-and-effect of each aspect.
